# Complete mitochondrial genome of *Stigmaeopsis miscanthi* (Acari: Tetranychidae)

**DOI:** 10.1080/23802359.2022.2055978

**Published:** 2022-05-11

**Authors:** Weijiu Tian, Tianci Yi, Daochao Jin, Yufeng Zhou

**Affiliations:** a Institute of Entomology, Guizhou University, Guizhou Provincial Key Laboratory for Agricultural Pest Management of the Mountainous Region, Scientific Observing and Experimental Station of Crop Pest in Guiyang, Ministry of Agriculture and Rural Affairs, Guiyang, PR China; b Guizhou Tea Research Institute, Guizhou Academy of Agricultural Science, Guiyang, China

**Keywords:** Mitogenome, *Stigmaeopsis miscanthi*, molecular phylogeny

## Abstract

Complete mitochondrial genome of *Stigmaeopsis miscanthi* was first reported for the genus *Stigmaeopsis* in Tetranychidae. The circular mitochondrial genome of *S. miscanthi* is 15,171 bp in length, with 80.4% AT content, involving 13 protein-coding genes, 22 transfer RNA genes, two ribosomal RNA, and main one of non-coding regions. The length of *16SrRNA* and *12SrRNA* gene is 973 bp and 636 bp, respectively. Seven PCGs started with ATT codon, and other PCGs started with ATG and ATA codons. Five PCGs stopped with TAA codon, and the other eight PCGs ended with incomplete stop codons (T). Phylogenetic analysis indicated that *S. miscanthi* branched with the genus *Panonychus*.

*Stigmaeopsis miscanthi* Saito 1990 (Acari: Tetranychidae) belongs to the family Tetranychidae, Acari, Arachnida. It was first described from Kyushu, Shikoku, and Okinawa in Japan (Saito 1990). *S. miscanthi* mainly inhabits on *Miscanthus grasses* (Poaceae), as other species of the genus *Stigmaeopsis*, has subsocial behavior such as communal living and nest building (Saito 1983, 2010, 2016; Mori et al. 1999). To our knowledge, there is currently no report on the complete mitochondrial genome of *Stigmaeopsis*.

The specimen of *S. miscanthi* was collected from Guiyang City (26°27′6″N, 106°39′26″E), Guizhou Province, China, in May 2021. This study got permissions from the Guizhou University Experimental Animal Ethics (reference number: EAE-GZU-2021-P012). The specimens (10 females and 10 males) were deposited in Institute of Entomology, Guizhou University, Guiyang, China (GCGU) with accession number 20210606J1, for further study (contact Dr. Jichun Xing, xingjichun@126.com). We extracted genomic DNA from adults of *S. miscanthi* via the rapid extraction kit for genomic DNA from tissue cells (Aidlab Biotechnologies Co., Ltd., Beijing, China) and short overlapping fragments sequenced by Sanger. The sequences were assembled and annotated using DNAstar, and analyzed and adjusted manually.

The phylogenetic tree was constructed by using the maximum-likelihood method for the software MEGA 7.0 and multiple sequence alignment was ClustalW (Kumar et al. 2016), based on the nucleotide sequence of 13 PCGs and two rRNAs of *S. miscanthi*. The mitogenomes of *S*. *miscanthi* and other 10 species of family Tetranychoidea were selected as ingroup, and two Eriophyoidea species were considered as outgroup.

The annotated mitochondrial genome sequence supporting the findings of this study has been openly available in GenBank under the accession number MZ726369 (https://www.ncbi.nlm.nih.gov/nuccore/MZ726369).

The obtained complete mitochondrial genome of *S. miscanthi* is 15,171 bp in length, and includes 13 protein-coding genes (PCGs), 22 tRNA genes, two rRNA genes, and one non-coding region. Seven of the 13 PCGs (*COI*, *ND1*, *ND2*, *ND3*, *ND5*, *ND4L*, and *Cytb*) begin with start codon ATT, and the other six genes begin with ATG or ATA as the start codon. Five PCGs (*COI*, *ND4L*, *Cytb*, *ND5*, and *ND6*) end with complete stop codons (TAA), and the other eight PCGs end with incomplete stop codons T. The lengths of 22 tRNA genes range from 42 bp (tRNA^Val^, tRNA^Ala^) to 70 bp (tRNA^Phe^), and *16S rRNA* possesses 973 bp and *12S rRNA* is 636 bp in length. In addition, the overall compositions of the bases A, G, C, and T are 33.0%, 11.0%, 8.6%, and 47.4%, respectively. The AT content is 80.4%, which is significantly higher than that the percentage of GC content (19.6%), showing an obvious A/T bias as the mitogenomes of other invertebrates.

The phylogenetic position of *S. miscanthi* shows the 11 Tetranychoidea species branch in a monophyletic group; *S. miscanthi* is closed related with *Panonychus citri* and *Panonychus ulmi* (Figure 1).

**Figure 1.**
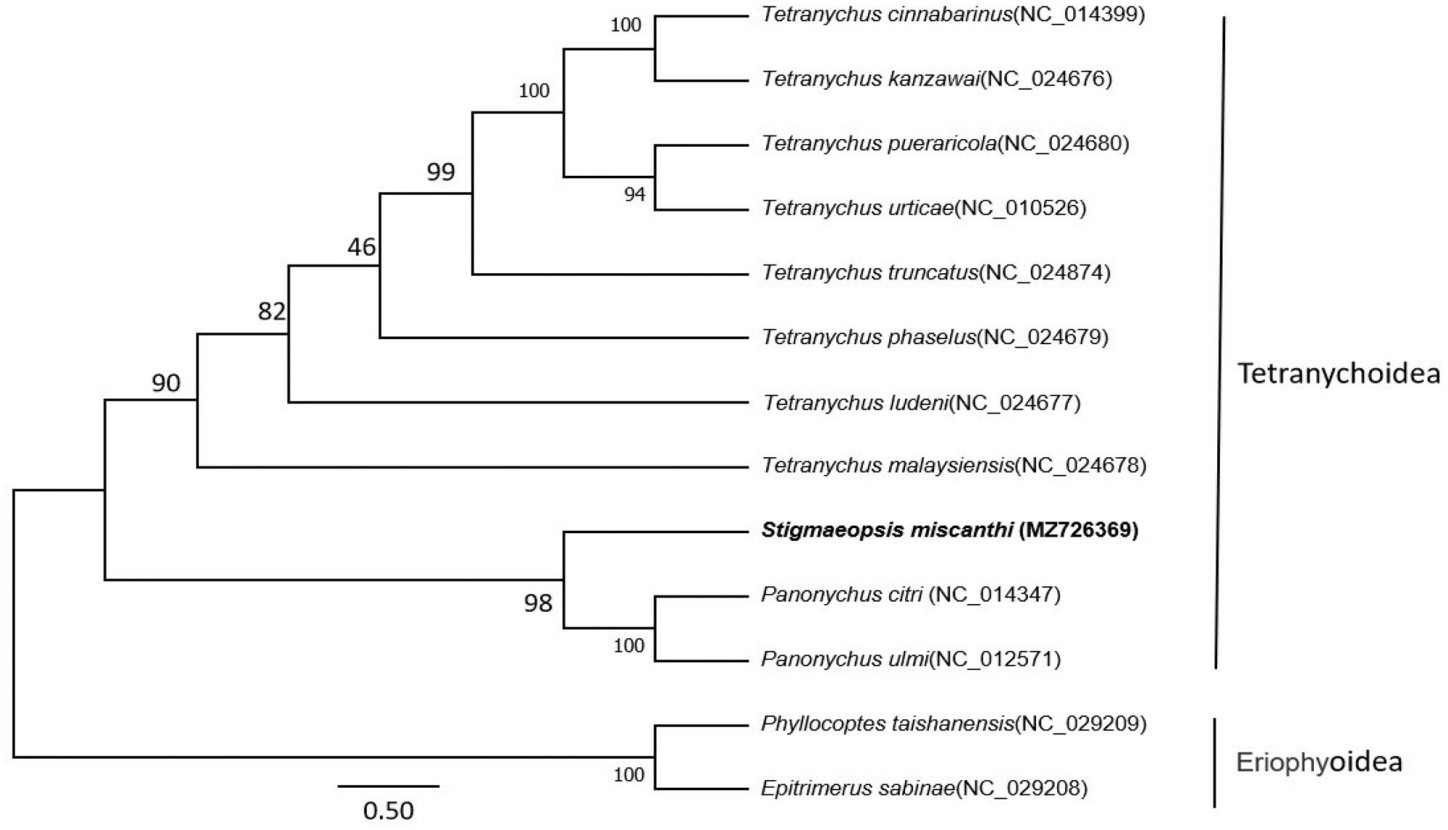
The phylogenetic tree was constructed using maximum-likelihood method with 1000 bootstrap replicates based on 13 protein-coding genes (PCGs)+two rRNAs of *S. miscanthi* and other 10 family Tetranychoidea species. Two Eriophyoidea species were selected as the outgroup. The GTR + G model was estimated as the best-fit substitution model according to the BIC standard in MEGA. The position of *Stigmaeopsis miscanthi* (whose mitogenome was determined in this study) is shown in bold. Posterior probabilities at correspondent nodes are shown in percentages. GenBank accession numbers for each species are shown in parentheses.

## Data Availability

The sequence data generated in this study were openly available in GenBank (https://www.ncbi.nlm.nih.gov/nuccore/MZ726369).
